# Development of a human peripheral blood *ex vivo* model for rapid protein biomarker detection and applications to radiation biodosimetry

**DOI:** 10.1371/journal.pone.0289634

**Published:** 2023-08-10

**Authors:** Leah Nemzow, Thomas Boehringer, Bezalel Bacon, Helen C. Turner

**Affiliations:** Center for Radiological Research, Columbia University Irving Medical Center, New York, New York, United States of America; Georgetown University, UNITED STATES

## Abstract

In the event of a widespread radiological incident, thousands of people may be exposed to a wide range of ionizing radiation. In this unfortunate scenario, there will be a need to quickly screen a large number of people to assess the amount of radiation exposure and triage for medical treatment. In our earlier work, we previously identified and validated a panel of radiosensitive protein biomarkers in blood leukocytes, using the humanized-mouse and non-human primate (NHP) models. The objective of this work was to develop a high-throughput imaging flow-cytometry (IFC) based assay for the rapid measurement of protein biomarker expression in human peripheral blood samples irradiated *ex vivo*. In this assay design, peripheral human blood samples from healthy adult donors were exposed to 0–5 Gy X-irradiation *ex vivo* and cultured for up to 2 days. Samples were stained with a cocktail of surface antigens (CD66b, CD20, and CD3), fixed and permeabilized, and intracellularly stained for BAX (Bcl-2-associated X) protein, used here as a representative biomarker. Samples were interrogated by IFC, and a uniform analysis template was created to measure biomarker expression in heterogeneous and specific leukocyte subtype populations at each time point. In this human blood *ex vivo* model, we show that within gated populations of leukocyte subtypes, B-cells are highly radiosensitive with the smallest surviving fraction, followed by T-cells and granulocytes. Dose-dependent biomarker responses were measured in the lymphocytes, B-, and T-cell populations, but not in the granulocytes, with dose-response curves showing increasing fold changes in BAX protein expression up to Day 2 in lymphocyte populations. We present here the successful use of this *ex vivo* model for the development of radiation dose-response curves of a candidate protein biomarker towards future applications of dose reconstruction and biodosimetry.

## Introduction

In the event of a disastrous large-scale unplanned exposure to ionizing radiation, such as the detonation of an improvised nuclear device (IND) or meltdown of a nuclear reactor, physical dosimeters are often not present among all those potentially exposed in the nearby vicinity, and thousands of people could be left without knowledge regarding their radiation exposure. Rapid and reliable dose estimation will be necessary to conduct early population triaging on a high volume of people to efficiently relieve the worried well and begin administering medical care to exposed individuals, thereby reducing the strain on medical resources and facilities. Post-exposure dose assessment techniques exist but are limited in a mass-casualty scenario. For example, clinical examinations of symptoms associated with prodromal acute radiation syndrome (ARS) require a high volume of trained personnel and will not feasibly allow for the inspection of all exposed individuals in a timely fashion [1]. Lymphocyte depletion kinetics require the establishment of early baseline counts and serial measurements, and a mass-screening scenario will likely not allow access to these samples [2]. Gold-standard cytogenetics requires transporting field samples to specialized facilities [3]. Thus, there exists a critical need for the development of novel radiation biodosimetry point-of-care (POC) devices that can quickly estimate patient-specific radiation dosage in large populations, days after exposure, without the need for skilled professionals or specialized facilities.

Biomarkers serve as useful diagnostic tools for triaging as they provide patient-specific information about the radiation-induced effects on biological functions which can be directly related to clinical consequences for the individual [4]. Since each biomarker may possess inherent unique characteristics with regard to dose range sensitivity, kinetics, and demographics, it will be important to identify and validate a panel of radiosensitive biomarkers that can be used to assess radiation exposure across the general population. This approach has been previously used in the development of gene [5–7] and plasma [8] protein biomarkers for radiation biodosimetry, yet studies of lymphocyte protein biomarkers for this purpose remain in their early stages of development. Protein biomarkers are particularly advantageous in a large-scale emergency response scenario due to their generally temporally persistent nature [9]. Previous work in our group identified a panel of candidate human lymphocyte intracellular radiosensitive protein biomarkers and subsequently validated these biomarkers for dose reconstruction in humanized mice and NHP models [10, 11]. Towards the goal of robust biomarker validation and performance evaluation in human blood, the objective of this work was to develop a simple high-throughput *ex vivo* whole blood culture model to rapidly measure radiosensitive biomarker expression in human peripheral blood up to two days post-radiation exposure.

**Fig 1:**
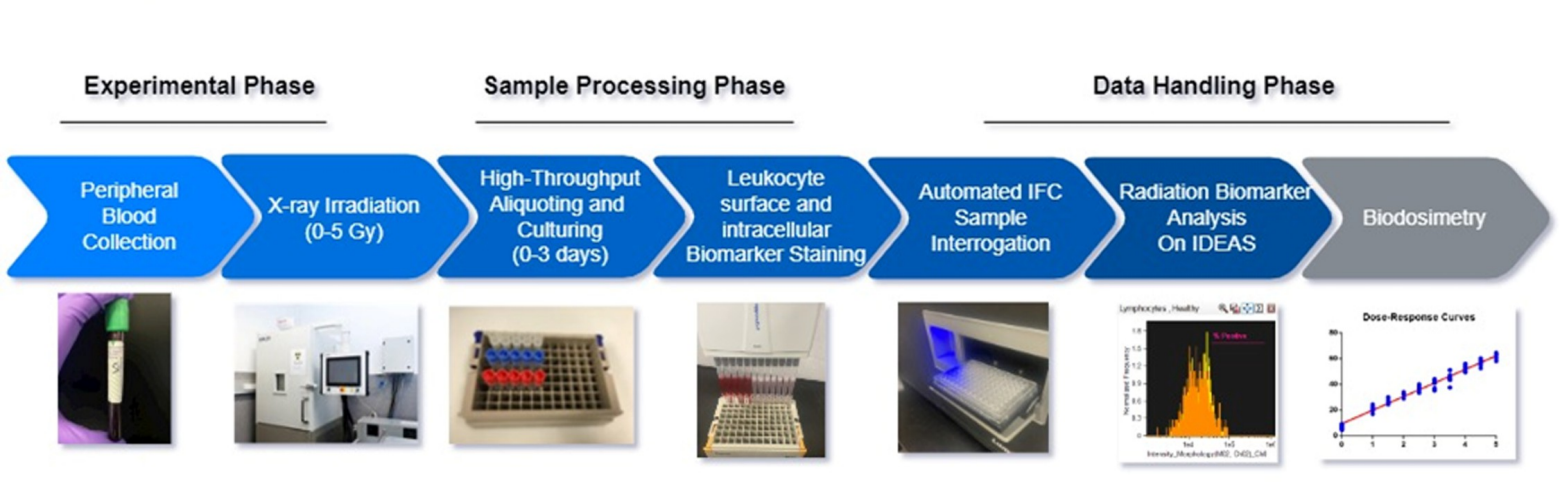
High throughput *ex vivo* assay for protein biomarker detection in human peripheral blood. **A) Experimental Phase.** Human peripheral blood samples were obtained from healthy donors by venipuncture and x-irradiated at ∼1Gy/min, 0-5Gy doses. **B) Sample Processing Phase.** x-irradiated human peripheral blood was aliquoted in small volumes of blood (100 °l) for cell culture in individual 1 mL Matrix^TM^ tubes with the 8×12 format rack. In a large-scale format using automated multi-channel pipettes, blood samples were labeled with CD66b, CD20, CD3 cell surface antibodies, lysed, fixed, and permeabilized, and stained with antibodies against biomarkers. **C) Data Handling Phase.** Stained and fixed blood leukocytes were then transferred with an automated multi-channel pipette into 96-well plates, and then interrogated and imaged by ImageStreamX (ISX) IFC auto-sampler system. A total of 3000 single and focused cells per sample were imaged on the ISX within 5 minutes. Protein Biomarker detection in leukocyte subtypes was measured and analysed in Image Data Exploration and Analysis Software (IDEAS^®^). Biomarker expression as a function of radiation dose in each donor was compiled and analysed by linear regression as a precursor for radiation biodosimetry and dose-prediction.

Here, we present the development of an *ex vivo* blood culture model for the rapid and high throughput detection of radiosensitive intracellular protein biomarkers using one of our previously identified radiation biomarker candidates, BAX, as a representative biomarker. BAX (Bcl-2-associated X protein) is a proapoptotic protein known to play a well-established role in radiation-induced cell death due to DNA damage, and its expression is upregulated by ionizing radiation [12–14]. **Fig 1** presents an overview of the high-throughput assay workflow which rapidly generates biomarker measurement in X-irradiated whole blood. Whole peripheral blood samples were exposed to 0–5 Gy X-ray doses, aliquoted in small volumes in a large-scale format, and cultured *ex vivo* for up to two days. Using a fluorescent immunoassay and automated Imaging Flow Cytometry (IFC), we identified leukocyte subpopulations within whole blood samples and quantified BAX protein expression and cell viability in leukocyte subtypes. Batch analysis of sample files from the IFC quickly generated a high volume of data that was used to create radiation dose-response curves for the BAX biomarker, which will serve as the basis for radiation biodosimetry.

## Materials and methods

### Human blood sampling and irradiation

Peripheral whole blood (≥ 5mL) from a total of 23 unique healthy human donors (7 males, 16 females, aged 22–63 years old, no X-ray exposure in the past 6 months, written consent obtained, approved by Columbia University IRB AAAS7621) was collected by venipuncture BD Vacutainers® with Sodium-Heparin (BD Biosciences 367878). Blood samples from each donor were aliquoted and mock irradiated or X-irradiated by X-RAD 320 biological irradiator (Precision X-Ray Inc., North Branford, CT) up to total doses of 1, 2, 3, 4, or 5 Gy, with the following conditions: custom house made filter: 1.5mmAl 0.25Cu 1.25Sn, 320 kVp, 12.5 mA, FSD 40, 0.95 Gy/min. Dose rate was validated with an ion chamber (Radcal® 10X6-6) before each sample irradiation.

### Peripheral blood culturing, staining, and fixation

Mock or X-irradiated blood samples were aliquoted (100 °l) into Matrix™ 1.0mL tubes (Thermo Fisher Scientific™ 3740TS, Waltham, MA) with 900 °l complete RPMI (15% FBS, 1% Pen-Strep) and processed immediately or cultured at 37°C, 5% CO_2_ for 1 or 2 days. Peripheral whole blood aliquots were surface stained for 20 minutes in the dark at room temperature with the following antibodies: CD66b-PE (mouse, monoclonal-G10F5, BioLegend 305105, 1:50, San Diego, CA), CD20-PE/Dazzle™ 594 (mouse, monoclonal-2H7, BioLegend 302347, 1:50), CD3-PE/Cy5 (mouse, monoclonal-HIT3a, BioLegend 300309, 1:50), and then lysed for 10 minutes at room temperature with eBioscience™ 1X RBC Lysis Buffer (Invitrogen, 00-4333-57). After washing in 1% BSA/PBS, sample aliquots were then fixed at 4°C using the Cytofix/Cytoperm™ Fixation/Permeabilization Solution Kit (BD Biosciences, 554714, Franklin Lakes, NJ). Leukocyte cells were washed with perm/wash buffer from the Cytofix/Cytoperm™ Fixation/Permeabilization Solution Kit and then intracellularly stained with BAX-AlexaFluor488 (mouse, monoclonal-2D2, BioLegend 633604, 1:200) for 1 hour in the dark, at room temperature. Samples were then washed twice with PBS and stored in 1ml PBS until sample acquisition. Antibodies were titrated to optimize staining index values and maximize spectral resolution.

### Viability staining and cell counting

Overall leukocyte viability data was obtained after incubation in RBC lysis buffer, as described above. Remaining leukocytes were stained with Acridine Orange/Propidium Iodide (AO/PI) viability dye (Logos Biosystems, F23001, South Korea), loaded into PhotonSlide™ (Logos Biosystems L12005), and automatically counted by LUNA-FL™ Dual Fluorescence Cell Counter (Logos Biosystems, L20001), as per manufacturer’s instructions.

### Imaging flow cytometry

#### Sample acquisition

Sample aliquots were centrifuged, and the stained and fixed cells were concentrated to a volume of 50°l. Focused and single cells (3,000) were acquired using the automated plate reader on the ImageStream MkII Imaging Flow Cytometer (Luminex Corporation, Austin, TX) at 40× magnification, 488 nm excitation laser, 200 mW. For compensation, cells stained with single fluorescence only were captured using the 488 nm laser with the brightfield and side scatter inactivated. The compensation coefficients were determined automatically by the compensation wizard and all captured images were analyzed within the IDEAS® software (Luminex ver. 6.2).

#### Analysis: Population selection and biomarker quantification

**Fig 2:**
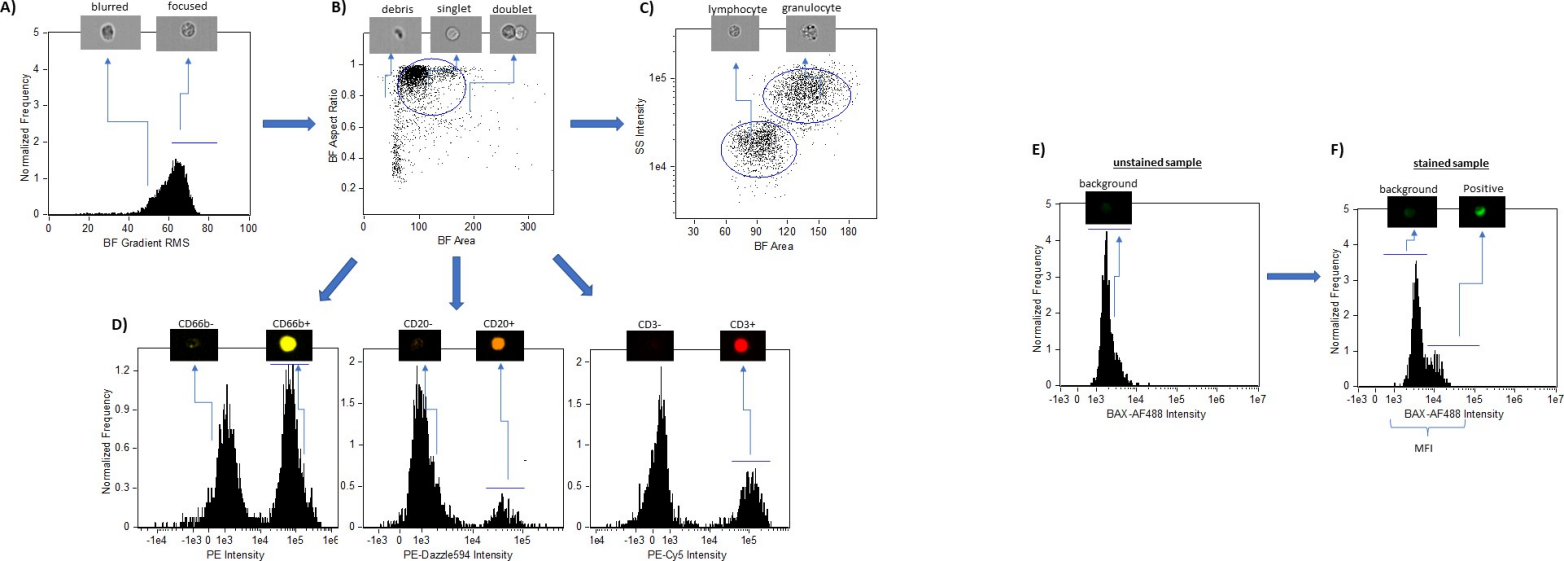
Representative analysis template for leukocyte identification and biomarker quantification on IDEAS®. **A) Gating focused cells**. The Gradient Root Mean Squared (RMS) feature identified focused cells in brightfield (BF) and elimination of blurred images. **B) Gating single cells**. BF Area versus BF Aspect Ratio permits bivariate plot allows for the selection of single cells, excluding doublets and debris. **C) Morphological identification of lymphocytes and granulocytes.** BF area versus SS intensity bivariate plot allowed for the gating of lymphocytes and granulocytes. **D) Immunophenotyping leukocyte subtypes. Granulocytes, B-cells, and T-cells were selected for by gating on CD66b+, CD20+ and CD3+ cells, respectively. E) Defining background**. Mean background signal intensity from several unstained sample is defined. **F) Using analysis template to measure biomarker expression in biomarker-stained samples.** Histogram of AlexaFluor 488 intensity for quantifying biomarker expression in heterogeneous, morphologically identified, and surface labeled populations, using MFI and % Positive metrics.

A uniform analysis template using Image Data Exploration and Analysis Software (IDEAS®, Luminex ver. 6.2) was created to select for leukocyte populations and define regions of biomarker measurements, as follows: Focused cells were selected by visually inspecting captured cell images and using the brightfield (BF) gradient root mean square (RMS) feature (**Fig 2A**). Single cells were gated using a bivariate plot of BF area versus BF aspect ratio, eliminating debris and doublets, creating the defined “heterogeneous” singlets leukocyte population (**Fig 2B**). For morphological selection of leukocyte subtypes, lymphocyte and granulocyte populations were gated using a bivariate plot of BF area versus fluorescence intensity of the side scatter (SS). Discrete event clusters with less BF area and SS intensity were defined as “lymphocytes”, and those with more BF area and SS intensity were defined as “granulocytes” (**Fig 2C**). For immuno-phenotypical selection of leukocyte subtypes, regions were set to gate to for positive fluorescence intensity of CD66b, CD20, and CD3 surface stains, defined as “(granulocytes) CD66b+”, “(B-cells) CD20+”, and “(T-cells) CD3+” (**Fig 2D**). To quantify non-specific background signal, fluorescence intensity on the 480–560 nm detector was examined in several unstained samples and a mean “background” region boundary was set in each gated population (**Fig 2E**). All intensity values above this “background” region boundary were defined as “Positive”. To measure biomarker expression in each gated leukocyte population, the uniform analysis template was applied to each biomarker-stained sample and automatically batch processed in IDEAS®**.** The Mean Fluorescence Intensity (MFI) of the biomarker, and percentage of cells that appeared in the “Positive” region (“% Positive”) of the biomarker fluorescence intensity were computed (**Fig 2F**).

### Statistical analysis

The differences in biomarker expression in fresh blood and blood cultured for 1 and 2 days within each subtype were analyzed by two-way ANOVA with Tukey’s multiple comparison test. The differences in biomarker expression in mock and 3 Gy X-irradiated cells were analyzed by multiple comparison paired Student’s T-tests, and the false discovery (FDR) approach (two-stage set-up method of Benjamini, Krieger, and Yekutieli) was used. Desired FDR (Q) was set to 1%. Two-tailed p values less than 0.05 were considered statistically significant. Simple Linear Regression was used to test if X-ray dose significantly predicted leukocyte viability/surviving fractions or biomarker fold changes; when values were normalized to those at 0 Gy, the regressions were constrained with y = 1. ANCOVA was performed to compare the slopes of the fitted regressions. Pearson product-moment correlation coefficients were computed to assess the relationship between loss of surviving fractions and biomarker fold changes. All statistical analyses were performed using GraphPad Prism (version 9.4.1; GraphPad Software, Inc., La Jolla, CA).

## Results

### Radiosensitivity of human leukocytes in the *ex vivo* blood culture model

**Fig 3:**
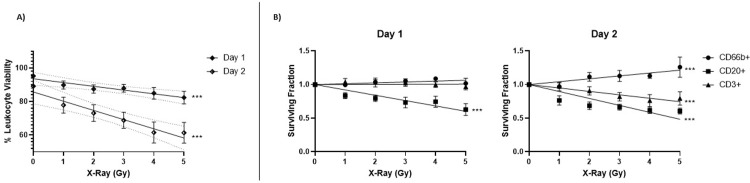
Radiosensitivity of leukocyte subtypes in the ex-vivo model. **A)** Percentage of leukocyte viability as measured by AO/PI staining and fluorescent cell counting on Day 1 and Day 2 post-irradiation, calculated as [number of live nucleated cells/number of total nucleated cells] (n = 12). Error bars represent ± SEM; p values reflect the significance for linear regression. **B)** Surviving fractions of gated populations of surface labeled leukocyte subtypes on Day 1 and Day 2 post-irradiation, calculated as [number of labeled events at dose x/number of labeled events at 0Gy] (n = 9). Data points for all subtypes at 0 Gy are found at Surviving Fraction 1.0, and the symbols are superimposed over each other. Error bars represent ± SEM; ***p < 0.001; p values reflect the significance for linear regression. Full regression analyses including regression equations, R^2^ values, and comparisons of slopes, can be found in S1 and S2 Tables.

To evaluate the *ex* vivo model in terms of cell death as a function of both X-ray dose and time, we examined leukocyte radiosensitivity as a measure of viability after radiation exposure. Propidium Iodide/Acridine Orange staining followed by automated cell counting on days 1 and 2 post 0–5 Gy X-irradiation show that leukocyte viability in our *ex vivo* model decreases in a radiation dose-dependent and time-dependent fashion (**Fig 3A)**. Linear regression analysis of leukocyte viability show that overall cell death does not reach a maximum at day 1, but rather continues increasing up to day 2, as indicated by higher slope value and a lower y-intercept as compared to 1-day post-irradiation (-2.248 vs -5.494 and 93.60 vs 85.71 on Days 1 and 2, respectively).

We also used the IFC method to examine cell death of different leukocyte subtypes within the cell culture population (**Fig 3B**). Numbers of surface-stained granulocytes, B- cells, and T-cells were quantified after each dose and time point and normalized to the levels measured in the pre-irradiated 0 Gy samples, generating surviving fraction values. At both timepoints, CD66b+ granulocytes showed apparent radioresistance with no change in surviving fractions across the tested dose range, whereas the surviving fractions of CD20+ B-cells and CD3+ T- cells decreased as a function of X-ray dose. The data show that CD3+ T-cell surviving fractions did not decrease in the Day 1 cultures but showed a significant (p < 0.001) dose dependent decrease by Day 2, whereas CD20+ B-cells showed a significant (p < 0.001) dose-dependent decrease in cell survival at Day 1 that further decreased by Day 2, indicating that the B- cells are more radiosensitive than T-cells.

As CD20+ B-cells comprise only about 3–10% of human peripheral blood cells, while CD3+ T-cells are more abundant with an estimated frequency of 10–25%, we speculate that the increased dose-dependent loss of CD3+ T-cells surviving fractions on day 2 likely accounts for the overall decreased viability on Day 2 versus Day 1 that is seen by AO/PI viability measurement (**Fig 3A)**. Taken together, the viability percentages in whole leukocyte populations up to 2 days after radiation exposure demonstrate a radiation dose and time-dependent model, while measurements of leukocyte subtype surviving fractions identify the extent to which each subpopulation contributes to overall radiosensitivity.

### Quantification of biomarker baseline levels of BAX in leukocyte subtypes

**Fig 4:**
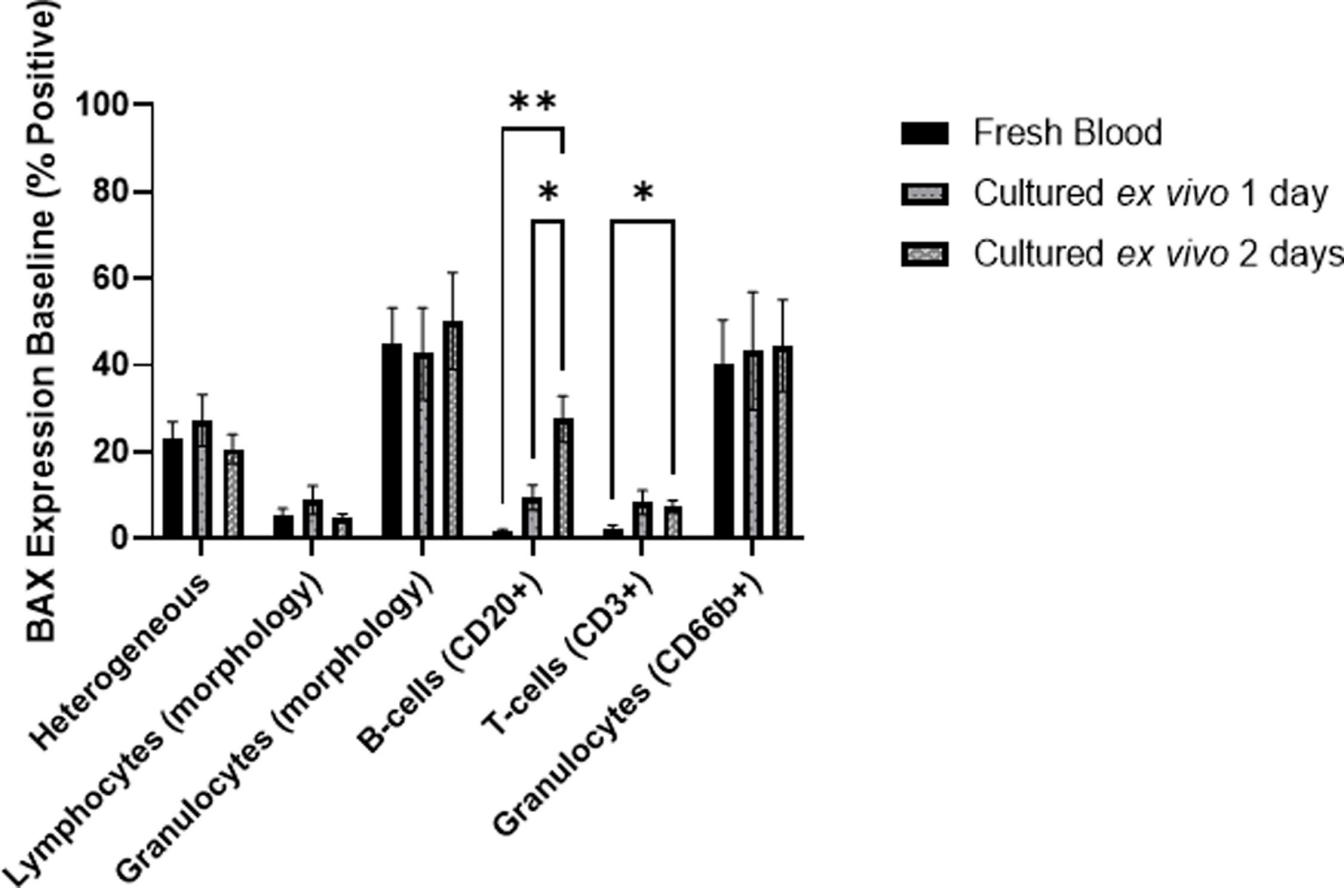
BAX baseline biomarker signal in leukocyte subtypes. Peripheral blood samples from healthy donors were either fixed and stained immediately after blood draw (“Fresh Blood”), or aliquoted and cultured as described in the methods for either 1 or 2 days. BAX biomarker expression in leukocyte populations from these paired samples was measured by IFC as described above. n = 10; error bars represent ± SEM; *p < 0.05; **p < 0.01; p values reflect the significance of two-way ANOVA.

Towards the development of an *ex vivo* culture model for measuring radiation-induced changes in biomarker expression, we first compared the baseline levels of BAX protein expression (% Positive) in non-irradiated leukocyte subtypes in fresh and cultured blood samples up to 2 days after exposure. **Fig 4** shows that in gated lymphocyte, CD3+ and CD20+ cell populations, BAX expression is very low in fresh blood, with a small significant increase in levels in CD20+ cells after 1 day in culture. However, by the second day in culture, both CD3+ and CD20+ populations show increases in BAX expression, with the CD20+ population showing a more significant increase in BAX expression, indicating increased sensitivity in this *ex vivo* model.

By contrast, the granulocyte population (gated by morphology or surface-labeled with CD66b+) show high baseline levels of BAX in all the fresh blood and cultured samples, with % Positive values in the range of ∼45–50%. The heterogeneous population which, comprised of single and focused cells from all the leukocyte sub-populations, also present high levels of BAX baseline levels, although substantially lower than the granulocyte populations (with % Positive values in the range of ∼20.5–23%), highlighting the contribution of CD3+ T-cells and CD20+ B-cells with very low BAX baselines.

### BAX biomarker expression in leukocyte populations is differentially induced by radiation

**Fig 5:**
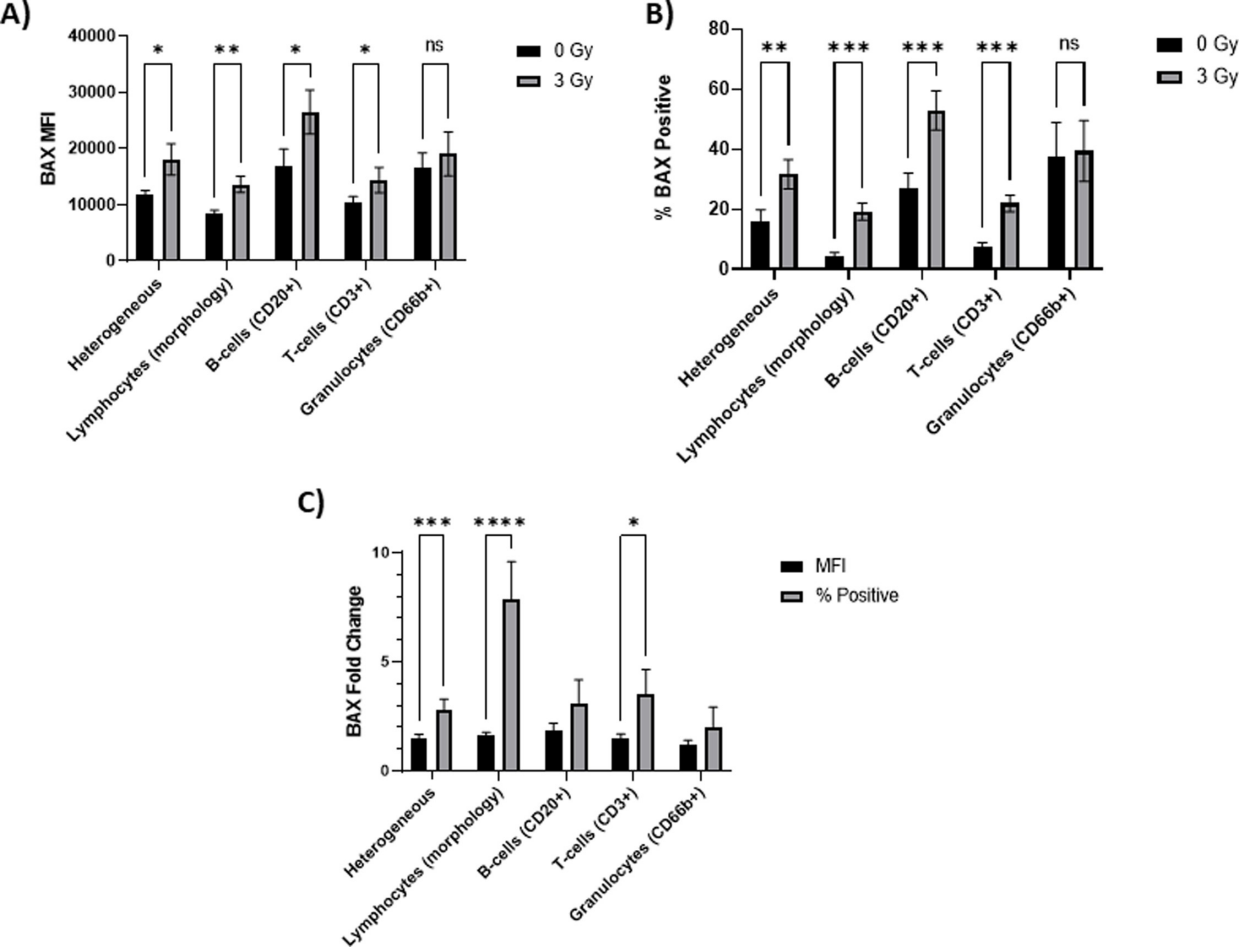
Quantifying BAX biomarker expression in leukocyte subtypes. Biomarker expression for BAX, 2 days post-irradiation was measured in heterogenous (all focused and single cells) and specific leukocyte subtypes using **A)** MFI or **B) % Positive**. C) Means were normalized to the 0 Gy means and resulting fold changes are reported. (n = 11). Data are expressed as mean ± SEM; Asterisks represent corrected p values in a multiple comparison paired t-test (*p < 0.05; **p < 0.01 and ***p < 0.001).

After evaluating the radiosensitivity of different leukocyte subtypes and baseline BAX expression in the *ex vivo* model up to 2 days in culture, we next measured BAX protein levels after X-irradiation. BAX expression, as measured using both MFI and % Positive metrics, was quantified in the different leukocyte subtypes, 1 and 2 days after exposure to 0 and 3 Gy X-ray. The 3 Gy dose point was chosen to test biomarker response in leukocyte subtypes because at 3 Gy exposures, we can measure a significant increase in BAX expression levels. Overall, MFI and % Positive measurements on Day 2 (**Fig 5**) were more amplified and more clearly differentially expressed between the subtype populations, as compared to Day 1 (which can be found in **S1 Fig**). In **Fig 5A and 5B** respectively, BAX biomarker expression was significantly increased in heterogeneous (p < 0.5; < 0.01), gated lymphocytes (p < 0.01;< 0.001), CD20+ B-cell (p < 0.05;< 0.001) and CD3+ T-cell (p < .05;< .001) populations after 3 Gy X-irradiation, while predictably, there was no increase in the granulocytes due to high baseline levels in the non-irradiated samples.

In **Fig 5C**, calculations of fold changes were used to compare levels of biomarker increase after 3Gy X-irradiation in leukocyte populations using the MFI and % Positive biomarker metrics. The results show that for all the leukocyte populations, the % Positive metric generated bigger assay windows compared to those from MFI, with means of fold changes ranging from 2.0 to 7.8 compared to a range of 1.2–1.8, respectively. Statistically significant increases in biomarker fold changes were measured in the heterogeneous (p < 0.001), gated lymphocytes (p < 0.0001), and CD3+ T-cell (p < 0.05) populations, with the largest biomarker fold change observed in the gated lymphocyte population (mean 7.8 vs 1.6). Although the CD20+ B-cells show the highest BAX % Positive mean (52.9%) after 3Gy in **Fig 5B**, the gated lymphocyte population produced the highest fold change primarily due to the low baseline levels of BAX protein in the non-irradiated control samples (mean % Positive of 4.2% compared to 27% in CD20+ B-cells), thus highlighting the importance of measuring biomarker increases in leukocyte populations with low baselines.

### Development of radiation dose-dependent biomarker curves

**Fig 6:**
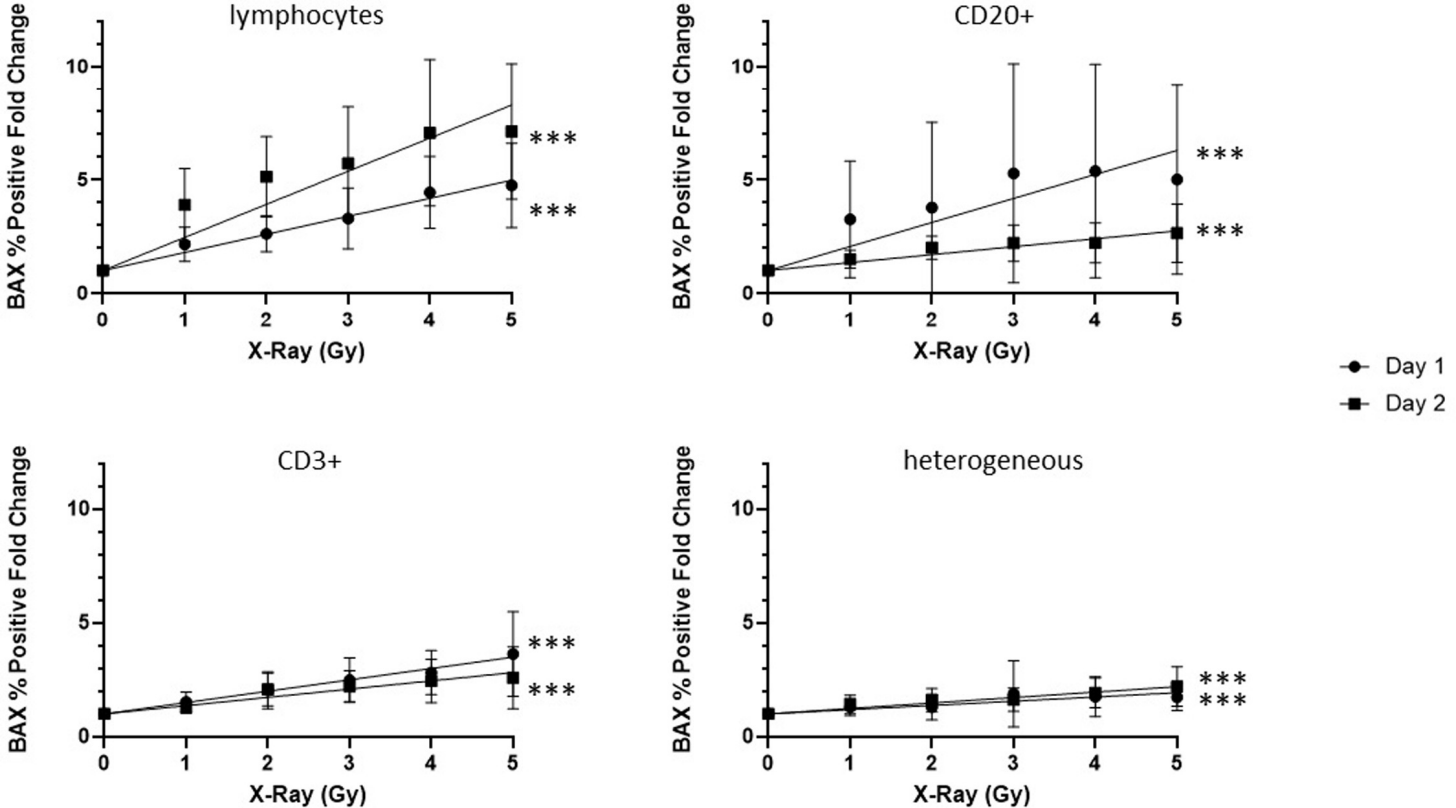
X-irradiation dose-curve: % positive BAX expression in lymphocyte populations on days 1 and 2 post-irradiation. Data represent mean fold changes of % Positive BAX expression (n = 6); Error bars represent ± SEM. Data points for all days at 0Gy are found at Fold Change 1.0, and the symbols are superimposed over each other. p values represent significance of linear regression (***p < 0.001). Full regression analyses including regression equations, and comparisons of slopes, can be found in S3 Table.

The % Positive metric was used to develop dose-response curves for the BAX biomarker in leukocyte populations up to 2 days after *ex vivo* X-irradiation. As seen in **Fig 6**, associations of BAX fold change and irradiated dose were examined by simple linear regressions in heterogeneous, gated lymphocyte, CD20+, and CD3+ populations. The data show that overall, regression models were statistically significant in all four leukocyte populations at both 1- and 2-days post-irradiation indicating that biomarker responses to X-irradiation are dose-dependent. Although X-ray dose significantly predicts increased BAX fold changes in each of the four populations examined here, the fitted regression models showed differential responses.

BAX % Positive fold changes were the most amplified in the gated lymphocytes which increased linearly with respect to dose, with the highest response following 4 Gy irradiation. The slope of the linear regression is higher on Day 2 than Day 1 (1.461 vs 0.7969), indicating a more robust biomarker response 2 days post-irradiation. Interestingly, linear regression analyses in CD20+ and CD3+ cells show the opposite trend where the fold changes in BAX are higher in the Day 1 cultures as opposed to the Day 2 (CD20+ slopes: Day 1 = 1.060, Day 2 = 0.3498; CD3+ slopes: Day 1 = 0.4998, Day 2 = 0.3637). This different response is likely due to lower baseline levels of BAX expression in CD20+ and CD3+ populations on Day 1 (as previously shown in **Fig 4**) which allow for the generation of higher fold changes. Overall, the mean baseline values for the three lymphocyte groups on Day 1 are comparable to each other, with similar fold change (ranging from 1 to 4-fold with increasing dose) and regression values, while the baselines values are more varied on Day 2, producing different fold change and regression values. Finally, the heterogeneous population shows a modest dose response on Day 1 (fold change means up to 1.7), and a slightly stronger response on Day 2 (fold change means up to 2.2), despite the strong presence of non-dose-responsive granulocytes in this population.

Simple bivariate linear regressions of fold changes were presented here to demonstrate the predictive association of X-ray dose on biomarker expression, as represented by the significant non-zero slope values, but they do not consider multiple variables or non-linear curves. The full dose-response curves (based on raw biomarker measurements) that will be created after the accumulation of more data and increased sample sizes will ultimately be modeled to account for variability in dose-response intercepts and slopes between different donors and non-monotonic or non-linear responses.

### Correlations between radiation-induced cell death and BAX expression in leukocyte subtypes

The simple linear regressions presented in **Figs 3 and 6** showed that X-ray dose is predictive of both, increased cell death and BAX expression, respectively. Comparisons of both linear regressions show that the subtypes and time points which show heightened radiosensitivity also show increases in BAX biomarker expression (CD20+ Days 1 and 2, CD3+ Day 2). Thus, we hypothesized that radiation-induced biomarker expression would increase proportionally with cell death, and that greater biomarker sensitivity would be seen in subtypes and time points with greater cell death. Pearson product-moment correlation coefficients were computed to test this hypothesis and determine whether there is a relationship between radiation-induced cell death and biomarker expression in this *ex vivo* blood culture model. **Table 1** summarizes the correlation between BAX fold changes and cell death as measured by the loss of the surviving fraction of CD66b+, CD20+ and CD3+ subtypes present in the cell cultures at 1 and 2 days after 0–5 Gy X-irradiation. Overall, these data show a significant positive correlation between BAX fold changes and cell death in CD20+ cells Day 1 and in both CD3+ and CD20+ cell populations on Day 2. CD66b+ cells show no positive correlation with these endpoints.

**Table 1:** Correlation of BAX expression fold changes and loss of surviving fraction in leukocyte subtypes.

		CD66b+	CD20+	CD3+
**Day 1**	Pearson Correlation	-0.402	0.917	0.5645
	Sig. (p, two-tailed)	0.4295	0.0285 *	0.2432
**Day 2**	Pearson Correlation	-0.0297	0.948	0.981
	Sig. (p, two-tailed)	0.9555	0.0264 *	0.0033 **

Means of BAX % Positive Fold Change and “lost cell fraction” in each stained leukocyte subtype at each X-ray dose were transformed in the natural log and plotted as scatter plots and analyzed for Pearson’s correlation (r). Lost cell fraction was calculated as [1- (surviving fraction of gated cells)]. n = 10. p values represent significance of Pearson product-moment correlation (*p < 0.05, **p < 0.01). In CD20+ 24 hours post X-irradiation, and both CD20+ and CD3+ 48 hours post X-irradiation, there was a positive correlation between the two variables.

## Discussion

A large-scale radiological emergency necessitates the need for rapid and high-throughput radiation biodosimetry testing of easily accessible biofluids (e.g., blood, urine, saliva) to enable swift triage and medical countermeasures. In our previous work, we have identified a panel of radiation-responsive protein biomarkers in human peripheral blood lymphocytes *in vivo* using the humanized moused model [10, 11]. Blood is an easily accessible biofluid for rapid collection after a large-scale radiological nuclear incident, but it is also a mixed material with many different components and cell types, which presents a challenge for robust biomarker detection in target leukocytes. We have successfully overcome this challenge by developing a high-throughput human blood *ex vivo* assay protocol that quickly and easily eliminates erythrocytes from whole blood and then uses the IFC platform to rapidly quantify immune-labeled intracellular protein biomarker signals in specific leukocyte populations. Here, we used BAX as a candidate dosimetry biomarker to develop our rapid and high throughput *ex vivo* blood culture model for quantifying radiosensitive protein biomarkers and building radiation dose-response curves in peripheral human blood leukocyte subpopulations after radiation exposure.

As part of designing an assay that measures radiation-induced changes of intracellular biomarkers in human blood, we evaluated the radiosensitivity of the model itself by examining a hallmark of ionizing radiation exposure: cell death [15, 16]. Radiation-induced apoptosis of blood lymphocytes is an established feature and biomarker of radiation exposure [17–19]. It has been well demonstrated that circulating lymphocyte populations in humans exponentially decrease after radiation exposure [20–23], and lymphocyte depletion kinetics are currently already used as a POC metric for assessing radiation exposure [24, 25]. Previous studies evaluating radiosensitivity of lymphocytes in human *ex vivo* models [26], as well as humanized mice [10] and radiotherapy contexts [27] have shown B-cells are highly radiosensitive, and to a greater degree than T-cells. Here, we examined the radiation-induced cell death in this *ex vivo* model by quantifying viability percentages and surviving fractions as functions of radiation dose. As expected, we observed that overall leukocyte viability declined in time- and dose- dependent manner **(Fig 3A)**. Specifically, we showed that within the surface-stained leukocyte subpopulations, CD66b+ granulocytes showed no dose-dependent cell loss, whereas the surviving fractions of lymphocyte cells were radiosensitive up to 2 days post X-irradiation. The CD20+ B-cells were the most radiosensitive subtype, showing loss of surviving fractions by 1-day post-exposure, while the CD3+ T-cells did not show cell death until Day 2. **(Fig 3B)**. Consistent with previous observations, the dose-dependent cell depletion measured here after irradiation validates our irradiation and cell culture model to study the dose response of radiosensitive biomarkers in human peripheral blood. Indeed, we found statistical correlations between radiation-induced BAX biomarker fold changes and cell death in B- and T- cells populations (**Table 1**), signifying the importance of a model design in which lymphocyte populations decline as a function of radiation dose.

Measurements of the BAX protein levels in specific leukocyte subtypes highlighted their unique responses to X-irradiation and culturing **(Figs 4–6)**. CD66b+ granulocytes showed high pre-irradiated biomarker baselines with no increase in BAX biomarker measurements after 3 Gy irradiation and are thus, not a useful target cell for measuring radiation dose-dependent BAX changes. Although CD20+ B- and CD3+ T-cells both showed statistically significant radiation-induced increases in BAX biomarker expression, the results show that CD20+ B-cells were more radiosensitive and showed the highest % BAX Positive fold change after radiation exposure (**Fig 5**). The large dose-dependent loss of B-cells after 2 days in culture limits their use in this *ex vivo* cell model as there is a practical limitation here of acquiring enough B-cells in each sample for adequate biomarker detection and statistical rigor. The data also show that radiation-induced BAX levels are highest at Day 1 and that baseline levels in non-irradiated samples dramatically increase with amount of time in culture thereby leading to a reduced amplification and dose-dependent fold changes by 2 days in culture (**Fig 6**). By comparison, measurements of BAX expression in CD3+ T-cells showed a more modest radiation-induced biomarker response without the limitation of low cell numbers and highly culture-sensitive biomarker baselines. As such, in the context of quantifying robust protein biomarker expression in an *ex vivo* culture days after radiation exposure, T-cells strike a more optimal balance in producing strong radiation-induced cell biomarker responses that are not compromised due to fast cell death kinetics and high biomarker baselines and serve as a strong cell target in the labeled leukocyte populations. Of note, CD20+ B-cells cells demonstrate low biomarker baselines in fresh blood with the ability to produce high biomarker signal after irradiation, and therefore warrant further studies in an *in vivo* context, when culturing limitations are no longer applicable.

As a versatile instrument, IFC is also capable of identifying leukocyte subpopulations by examining morphological features of acquired cells, without the need for surface labels, which further accelerates and simplifies the protocol. We directly compared biomarker performance in these morphologically gated lymphocyte populations to that in surface-labeled B-/T-cell populations and discovered that the gated lymphocytes showed the highest fold changes of BAX expression (**Fig 5C**), as well as the strongest dose-dependent increase after 2 days post-irradiation **(Fig 6)**. Yet, despite this amplified biomarker signal in this unlabeled lymphocyte population, the disadvantage of this approach is that gated lymphocytes showed morphological changes in brightfield area and side scatter parameters upon *ex vivo* irradiation and culturing (representative example seen in **S2 Fig**), thereby necessitating an additional level of dynamic input to accurately select lymphocytes. No such modifications are generally needed to define a surface labeled population, which would be a favorable feature of a POC device equipped to measure fluorescent signal and would allow the assay to transition to the mass casualty field setting. The heterogeneous population, uniquely, is a label-free population that also does not require any morphological information to identify it. While the dose curve in this population showed a lower biomarker response, this population might also serve as a useful target to measuring biomarker signal in a POC device.

Although the observations reported here demonstrate the use of one representative biomarker in the context of radiation exposure, this *ex vivo* assay and model design can be adapted for rapid protein biomarker measurements in many other contexts. Firstly, other radiation biomarkers may be tested; our proteomics study revealed 6 top-ranked radiation biomarker candidates found in humanized mice that included BAX [11], and further studies in our group will continue to validate these other biomarker candidates in human peripheral blood. Secondly, human leukocyte protein biomarkers that are responsive to other genotoxic and cytotoxic agents (such as chemotherapeutic agents, or drug side-effects) can be evaluated in this *ex vivo* blood culture model before attempting clinical trials *in vivo*. Thus, this *ex vivo* blood culture model potentially can also be used in a variety of drug testing and treatment planning contexts.

In summary, we show the successful use of this *ex vivo* blood culture model in measuring protein biomarkers to develop radiation dose response curves that will provide the basis for radiation biodosimetry and dose reconstruction in the event of a potential exposure to an unknown dose. We expect to continue to use this high-throughput assay for the validation of dose and time kinetics of our protein biomarker panel *in vitro* (including an increased range of dose and time-points), as well as extend the work to test the top-ranked biomarkers as part of a demographic study to determine whether potentially confounding factors (such as age, sex, race, smoking status, pre-existing medical conditions) might influence biomarker measurements and identify possible inter-individual variabilities. The leukocyte viability and biomarker kinetics observed here in this *ex vivo* model might differ in an *in vivo* context. Importantly, the inclusion of baseline biomarker measurements in non-irradiated fresh blood samples provides important information to assess biomarker variability *in vivo* in non-exposed individuals across the demographics. Additionally, these *ex vivo* biomarker validations will be conducted in tandem with FDA approved animal models and human patients receiving clinical treatments to evaluate and compare biomarker dose responses in various physiological and non-physiological contexts. Taken together, the radiation dose curves generated in these future studies will be used to develop mathematical models for estimating radiation dose.

## Supporting information

S1 Fig Quantifying BAX biomarker expression in leukocyte subtypes on day 1. Biomarker expression for BAX, 1 days post-irradiation was measured in heterogenous and specific leukocyte subtypes using A) MFI or B) % Positive. C) Means were normalized to the 0 Gy means and resulting fold changes are reported. (n = 11). Data are expressed as mean ± SEM; Asterisks represent corrected p values in a multiple comparison paired t-test (*p < 0.05; **p < 0.01 and ***p < 0.001).
(TIF)

S2 Fig Morphological identification of leukocyte types. Representative example of shifting scatter plots after culturing/irradiation. If the same gating coordinates that are used to identify lymphocytes in fresh blood are used in cultured blood, a large portion of lymphocytes would be missing from the gates. CD3+ stained cells are shown to illustrate the theoretical proper gating of morphologically identified lymphocytes.
(TIF)

S1 Table Simple linear regression analysis for Fig 3A. (XLSX)

S2 Table Simple linear regression analysis for Fig 3B. (XLSX)

S3 Table Simple linear regression analysis for Fig 6. (XLSX)
