# Supplementary figures and images for: Development of a human peripheral blood ex vivo model for rapid protein biomarker detection and applications to radiation biodosimetry
Source: PLoS One. 2023 Aug 10;18(8):e0289634. doi: 10.1371/journal.pone.0289634 (PMC10414586; doi:10.1371/journal.pone.0289634)

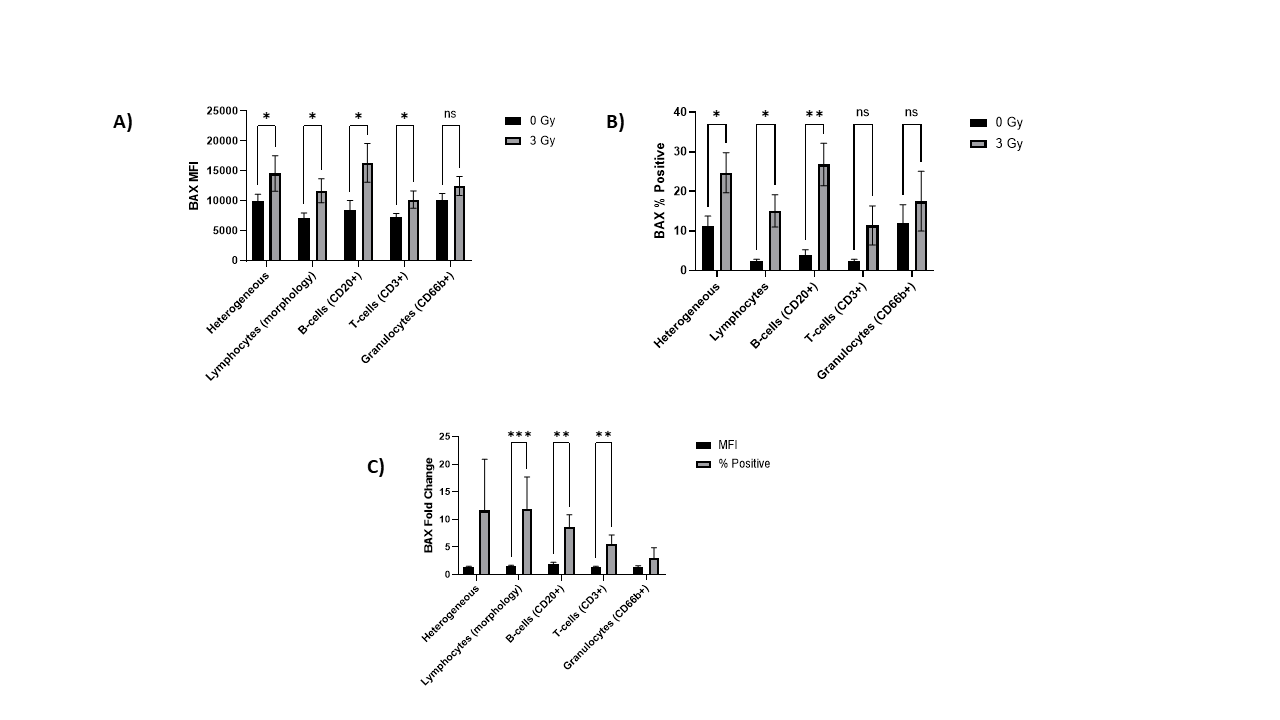

Supplement: S1 Fig — Biomarker expression for BAX, 1 days post-irradiation was measured in heterogenous and specific leukocyte subtypes using A) MFI or B) % Positive. C) Means were normalized to the 0 Gy means and resulting fold changes are reported. (n = 11). Data are expressed as mean ± SEM; Asterisks represent corrected p values in a multiple comparison paired t-test (*p < 0.05; **p < 0.01 and ***p < 0.001). (TIF) [file pone.0289634.s001.tif]

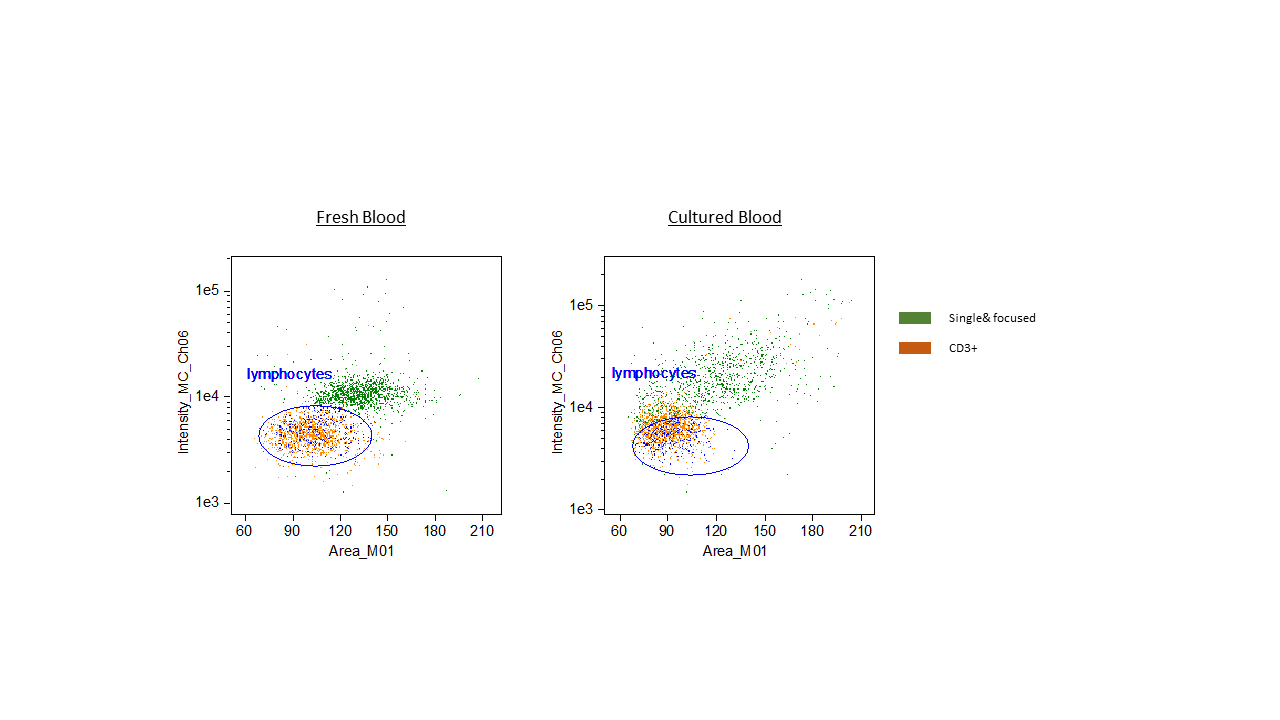

Supplement: S2 Fig — Representative example of shifting scatter plots after culturing/irradiation. If the same gating coordinates that are used to identify lymphocytes in fresh blood are used in cultured blood, a large portion of lymphocytes would be missing from the gates. CD3+ stained cells are shown to illustrate the theoretical proper gating of morphologically identified lymphocytes. (TIF) [file pone.0289634.s002.tif]
